# CC chemokine ligands in patients presenting with stable chest pain: association with atherosclerosis and future cardiovascular events

**DOI:** 10.1007/s12471-016-0884-9

**Published:** 2016-08-29

**Authors:** M. O. Versteylen, M. Manca, I. A. Joosen, D. E. Schmidt, M. Das, L. Hofstra, H. J. Crijns, E. A. Biessen, B. L. Kietselaer

**Affiliations:** 1 Cardiovascular Research Institute Maastricht (CARIM)/Department of Cardiology, Maastricht University Medical Center, Maastricht, The Netherlands; 2 Department of Pathology, Maastricht University Medical Center, Maastricht, The Netherlands; 3 Department of Cardiology, Maastricht University Medical Center, Maastricht, The Netherlands; 4 Cardiovascular Research Institute Maastricht (CARIM)/Department of Radiology, Maastricht University Medical Center, Maastricht, The Netherlands; 5 Cardiology Center Netherlands, Utrecht, The Netherlands; 6 Department of Cardiology and Radiology, Maastricht University Medical Center, Maastricht, The Netherlands

**Keywords:** CC chemokine ligands, Cardiac computed tomographic angiography, Coronary artery disease, Cardiac events, Prognosis

## Abstract

**Background:**

CC chemokine ligands (CCLs) are elevated during acute coronary syndrome (ACS) and correlate with secondary events. Their involvement in plaque inflammation led us to investigate whether CCL3-5-18 are linked to the extent of coronary artery disease (CAD) and prognostic for primary events during follow-up.

**Methods:**

We measured CCL3-5-18 serum concentrations in 712 patients with chest discomfort referred for cardiac CT angiography. Obstructive CAD was defined as ≥50 % stenosis. The extent of CAD was measured by calcium score and segment involvement score (number of coronary segments with any CAD, range 0–16). Patients were followed up for all-cause mortality, ACS and revascularisation, for a mean 26 ± 7 months.

**Results:**

Patients with obstructive CAD had significantly higher CCL5 (*p* = 0.02), and borderline significantly elevated CCL18 plasma levels as compared with patients with <50 % stenosis (*p* = 0.06). CCL18 levels were associated with coronary calcification (*p* = 0.002) and segment involvement score (*p* = 0.007). Corrected for traditional risk factors, only CCL5 provided independent predictive value for obstructive CAD: odds ratio (OR) 1.27 (1.02–1.59), *p* = 0.04. CCL5 provided independent predictive value for primary events during follow-up: OR 1.62 (1.03–2.57), *p* = 0.04.

**Conclusions:**

While CCL18 serum levels correlated with extent of CAD, CCL5 demonstrated an independent association with the presence of obstructive CAD, and occurrence of primary cardiac events.

## Introduction

Atherosclerosis is generally regarded as a dyslipidaemic disorder with a strong inflammatory character [1]. This inflammation process is in part directed by chemokines, which play a role in mediating leukocyte recruitment to sites of injury, vascular smooth muscle cell proliferation, neovascularisation and platelet activation [2, 3]. Among others, CC chemokine ligand (CCL) 3, 5 and 18 have been detected in human atherosclerotic lesions [4, 5].

In addition, CCL3, 5 and 18 have been associated with acute coronary syndrome (ACS) and refractory unstable angina pectoris (UAP) in several prospective studies. In patients with UAP, CCL5 and 18 were transiently raised during UAP and indicative of refractory syndromes [6]. CCL3 levels were significantly elevated in patients with acute myocardial infarction (AMI) as compared with controls, as well as transiently elevated in UAP patients. In addition, CCL3 elevation was associated with future ACS [7]. Also, in a larger cohort of patients presenting with ACS, CCL3, 5 and 18 were independently associated with fatal events during follow-up [8]. Moreover, macrophage expression of CCL18 in patients with atherosclerosis was significantly higher as compared with controls, suggesting CCL18 has a role in atherosclerotic plaque formation [9]. Nevertheless, several other studies investigating different populations reported no association between CCL5 and future cardiovascular events [10–12].

**Fig. 1:**
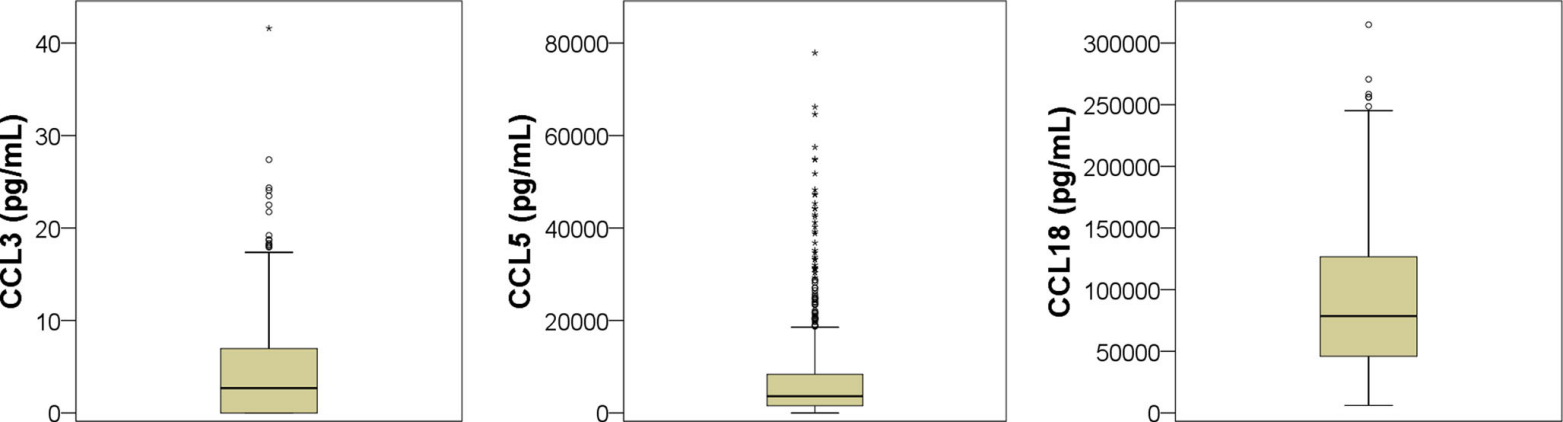
Median concentrations of CCL3, CCL5 and CCL18

Although CCL3, 5 and 18 might contribute to plaque formation, and could therefore represent attractive therapeutic targets, evidence is conflicting and their association with presence and extent of coronary artery disease (CAD) is not well understood. We hypothesised that CCL3, 5 and 18 are associated with both CAD and cardiac events in patients with stable chest pain.

The goal of this study was therefore to address the relationship between CCL3, 5 and 18 and the presence and extent of CAD as defined by coronary computed tomography angiography (CCTA) in a cohort of patients with stable chest pain. In addition, the association of CCL3, 5 and 18 with primary cardiac events during follow-up was investigated.

## Methods

### Study population

We analysed a cohort of patients who were referred from the outpatient clinic for CCTA to rule out CAD between January 2008 and June 2010. Included were patients who underwent both a calcium score scan and CT angiography. In total 1096 patients were scanned. Excluded were 117 patients who had an inconclusive CCTA, and 95 patients because of missing clinical data. Additionally, 172 blood samples were haemolytic and were excluded from this analysis. Eventually, 712 patients participated in this study and plasma was screened for CCL3, 5 and 18 levels. All patients signed informed consent. The study protocol conforms to the ethical guidelines of the 1975 Declaration of Helsinki, as reflected in approval by the Institutional Review Board and Ethics Committee (MEC 08-4-057).

### CCTA acquisition

CCTA was performed using a 64-slice multidetector row computed tomography scanner (Brilliance 64; Philips Healthcare, Best, the Netherlands) with a 64 × 0.625 mm slice collimation and a rotation time of 420 ms. In addition, a native scan using 120 kV and 3 mm slice thickness was performed to determine the coronary calcium score (Agatston score). The mean total estimated radiation dose was calculated by multiplying the dose-length product by the conversion factor of 0.014 mSv/mGy/cm for the thorax [13].

### CCTA assessment

The Agatston method was used to quantify the coronary calcium score [14]. CT angiograms were independently analysed by a cardiologist and a radiologist, both experienced in reading cardiac CT and blinded to clinical information, using the American Heart Association 16 coronary segments model [15]. In case of disagreement, consensus was reached by discussion between the two readers. The degree of stenosis was defined visually and a lesion severity score was calculated as follows: no lesion (score 0), mild diameter stenosis (<50 %, score 1), moderate (50–70 %, score 2) and severe diameter stenosis (>70 %, score 3). This resulted in a lesion severity score ranging from 0–3 for each individual patient. The presence of one or more lesions with ≥50 % stenosis was regarded as significant CAD. In addition, the segment involvement score was defined as the total number of coronary segments with detectable CAD (range 0–16) [16].

### Biomarker assessment

Blood samples were collected by ethylenediaminetetraacetic acid (EDTA) coated Vacutainer venipuncture just before the scan. Samples were kept at 4 °C and centrifuged at 4100 rpm for 10 minutes, and then stored at −80 °C until analysis, all within two hours. High-density lipoprotein cholesterol was measured using Synchron LX20 (Beckman Coulter). Low-density lipoprotein cholesterol was calculated using the Friedewald equation, or Cobas Mira Plus (Roche Diagnostics). High-sensitivity C‑reactive protein (hsCRP) was measured on BN ProSpec using the CardioPhase hsCRP assay (Siemens Diagnostics).

#### CCL assessment

Baseline blood specimens were collected from storage in July 2012. The sera were snap frozen and stored at −70 °C until further analysis and underwent only one freeze-thawing cycle. Baseline serum levels of CCL3/MIP-1α, CCL5/RANTES and CCL18/PARC were determined by commercially available ELISA kits from R&D systems (Abingdon, United Kingdom; Human CCL3/MIP-1 alpha Quantikine ELISA Kit, ≤8.9 % intra-assay variation, Human CCL 5/RANTES DuoSet and Human CCL18/PARC DuoSet). CCL3 ELISA was performed according to the manufacturer’s instructions. For CCL5 measurements, 0.75 μg/ml capture antibody and 10 ng/ml detection antibody were used, for CCL18 we used 1.5 μg/ml capture antibody and 200 ng/ml detection antibody.

## Follow-up

Patients were followed up for the occurrence of all-cause mortality, ACS (including myocardial infarction) and revascularisation procedures based on conventional coronary angiography including percutaneous coronary intervention (PCI) and coronary artery bypass grafting (CABG), for a mean of 26 ± 7 months. Electronic patient records were checked, ACS was defined as typical angina pectoris and troponin T elevation (>0.01 μg/l) and ST-segment elevation/depression of ≥1 mm, or at least two of these symptoms together with invasive angiographic confirmation of a culprit lesion [17]. Further, we censored follow-up after the first endpoint so that the recorded ACS was not a complication of revascularisation therapy.

## Statistics

Data were analysed using SPSS 22.0 (SPSS Inc., Chicago, IL, USA). Continuous variables were presented as mean ± standard deviation or median and interquartile range, whether they were of normal or nonparametric distribution, respectively. Differences were assessed using Student’s t‑test for normally distributed data, or Kruskal-Wallis test for nonparametric distributions. Proportions (%) were used for categorical values, in which differences were assessed using Fisher’s exact test. In regression models, base-10 logarithmic transformation was used in order to include continuous variables with a non-normal distribution. All *p*-values were two-sided, and a *p*-value below 0.05 was considered statistically significant.

## Results

### Study population

The mean age of the study population was 56 ± 11 years, 398 (56 %) were males. The indication for CCTA was typical chest pain in 88 (12 %), atypical in 310 (44 %) and non-anginal complaints in 314 (44 %). The mean radiation dose was 5.6 ± 4.5 mSv. The median concentration of CCL3 was 2.7 (0.0–7.0) pg/ml, for CCL5 it was 3614 (1547–8360) pg/ml and for CCL18 it was 78,553 (45,906–126,716) pg/ml (Fig. 1). Baseline characteristics are further described in Table 1.

**Table 1:** Baseline characteristics

	<50 % stenosis	≥50 % stenosis	*p*-value
	*n* = 509	*n* = 203	–
Age (years)	54.7 ± 10.2	60.9 ± 9.3	**<0.001**
Male gender	266 (52.3 %)	132 (65.0 %)	**0.002**
Smoking	107 (21.0 %)	62 (30.5 %)	**0.008**
Diabetes mellitus	41 (8.1 %)	17 (8.4 %)	0.88
Family history	187 (36.7 %)	85 (41.9 %)	0.23
Systolic blood pressure (mm Hg)	140 ± 17	147 ± 20	**<0.001**
BMI (kg/m^2^)	26.9 ± 4.1	27.4 ± 4.4	0.14
LDL cholesterol (mmol/l) HDL cholesterol (mmol/l) Statin use	3.3 ± 1.0 1.3 ± 0.5 167 (33.0 %)	3.3 ± 1.2 1.3 ± 0.8 100 (49.5 %)	0.67 0.51 **<0.001**
Typical chest pain	59 (11.6 %)	29 (14.3 %)	0.32
Calcium score	0 (0–24.5)	201 (53.5–455)	**<0.001**
Involvement score	0 (0–2)	5 (3–7)	**<0.001**
CCL3 (pg/ml)	2.5 (0.0–7.0)	2.8 (0.4–7.0)	0.37
CCL5 (pg/ml *10^2^)	33.4 (14.5–77.0)	40.7 (18.5–109.1)	**0.02**
CCL18 (pg/ml *10^2^) hsCRP (mg/dl)	747.1 (450.5–1241.3) 1.4 (0.7–1.4)	885.1 (502.5–1331.8) 1.7 (0.9–4.1)	0.06 **0.02**

Values are presented as numbers (%), means ± standard deviation or medians (IQR). Significant *p*-values are in bold. *BMI* body mass index, *CCL CC* chemokine ligand, *hsCRP* high-sensitive C‑reactive protein

### Association CCL with CAD

The serum concentration of CCL5 was significantly higher in patients with ≥50 % coronary stenosis, as compared with patients with <50 % stenosis (Table 1). CCL18 was associated with the calcium score and the segment involvement score (Table 2). As is shown in Table 3, only CCL5 provided independent information to predict a ≥ 50 % coronary stenosis when corrected for traditional risk factors.

**Table 2:** Univariable regression analysis to predict for extent of CAD

Coronary calcium score (log-transformed)			
	B-value	95 % CI	*p*-value
Age (years)	0.44	0.04–0.05	**<0.001**
Male gender	0.14	0.15–0.47	**<0.001**
Smoking	0.05	−0.05–0.33	0.15
Diabetes mellitus	0.10	0.12–0.70	**0.006**
Family history	0.01	−0.14–0.20	0.72
Systolic blood pressure (mm Hg)	0.23	0.01–0.02	**<0.001**
BMI (kg/m^2^)	0.06	−0.00–0.04	0.09
LDL cholesterol (mmol/l) HDL cholesterol (mmol/l) Statin use	−0.05 −0.01 0.19	−0.13–0.02 −0.16–0.12 0.27–0.59	0.17 0.75 **<0.001**
Typical chest pain	−0.01	−0.28–0.20	0.75
Lg10 CCL3	0.04	−0.09–0.28	0.33
Lg10 CCL5	−0.02	−0.11–0.07	0.60
Lg10 CCL18 Lg10 hsCRP ≥50 % stenosis	0.12 0.03 0.57	0.16–0.73 −0.12–0.25 1.23–1.52	**0.002** 0.50 **<0.001**
Involvement score (log-transformed)			
	B-value	95 % CI	*p*-value
Age (years)	0.38	0.01–0.02	**<0.001**
Male gender	0.20	0.09–0.19	**<0.001**
Smoking	0.09	0.01–0.13	**0.02**
Diabetes mellitus	0.10	0.03–0.22	**0.01**
Family history	0.03	−0.03–0.07	0.44
Systolic blood pressure (mm Hg)	0.23	0.00–0.01	**<0.001**
BMI (kg/m^2^)	0.03	−0.00–0.01	0.39
LDL cholesterol (mmol/l) HDL cholesterol (mmol/l) Statin use	0.00 −0.01 0.19	−0.02–0.03 −0.05–0.04 0.09–0.19	0.94 0.80 **<0.001**
Typical chest pain	−0.00	−0.08–0.08	0.97
Lg10 CCL3	0.04	−0.03–0.10	0.25
Lg10 CCL5	−0.03	−0.04–0.02	0.46
Lg10 CCL18 Lg10 hsCRP ≥50 % stenosis	0.10 0.03 0.66	0.03–0.22 −0.04–0.08 0.47–0.55	**0.007** 0.56 **<0.001**

Univariable linear regression model. *BMI* body mass index, *CCL* CC chemokine ligand, *hsCRP* high-sensitive C‑reactive protein

**Table 3:** Multivariable logistic regression analysis to predict for CAD (≥50 % stenosis)

	OR	95 % CI	*p*-value
*Model 1*			
Lg10 CCL3	1.01	0.67–1.53	0.96
Lg10 CCL5	1.23	0.99–1.53	0.06
Lg10 CCL18	1.03	0.55–1.93	0.92
*Model 2*			
Lg10 CCL3	0.96	0.62–1.47	0.84
Lg10 CCL5	1.27	1.02–1.59	**0.04**
Lg10 CCL18	1.06	0.56–2.03	0.86

Model 1: corrected for age and gender; Model 2: corrected for age, gender, diabetes mellitus, BMI, smoking, family history, systolic blood pressure and total cholesterol. *CAD* coronary artery disease, *CCL CC* chemokine ligand, *OR* odds ratio, *CI* confidence interval

### CCL concentrations and events

During a mean follow-up of 26 months, a total of 51 events occurred (3 all-cause mortality, 13 ACS, 10 CABG and 25 PCI). Patients who developed an event during follow-up were more often males, smokers and had higher calcium and segment involvement scores. Also, their median CCL5 concentration was higher (Table 4). When corrected for traditional risk factors in multivariable regression analysis, CCL5 concentration showed independent predictive value for the occurrence of an event during follow-up (Table 5, model 2). When also corrected for the extent of CAD (adding the segment involvement score, model 4), the independent prognostic value of CCL5 was still present. However, when correcting for the presence of significant stenosis (≥50 % stenosis, model 3), the significant prognostic value of CCL5 disappeared.

**Table 4:** Difference between patients developing an event and patients who do not develop an event

	Patients without event	Patients with event	*p*-value
	*n* = 661	*n* = 51	–
Age (years)	56 ± 10	58 ± 11	0.29
Male gender	361 (54.6 %)	37 (72.5 %)	**0.01**
Smoking	147 (22.2 %)	22 (43.1 %)	**0.002**
Diabetes mellitus	52 (7.9 %)	6 (11.8 %)	0.29
Family history	252 (38.1 %)	20 (39.2 %)	0.88
Systolic blood pressure (mm Hg)	142 ± 19	143 ± 20	0.67
BMI (kg/m^2^)	27 ±4	27 ±4	0.81
Total cholesterol (mmol/l) HDL cholesterol (mmol/l) LDL cholesterol (mmol/l) Statin use	5.4 ± 1.1 1.3 ± 0.6 3.3 ± 1.0 244 (37.1 %)	5.3 ± 1.3 1.2 ± 0.4 3.3 ± 1.2 23 (45.1 %)	0.61 0.10 0.79 0.29
Typical chest pain	81 (12.3 %)	7 (13.7 %)	0.67
Calcium score	4.6 (0.0–103.1)	181.0 (20.6–458.2)	**<0.001**
Involvement score	1.0 (0.0–3.0)	5.0 (2.0–7.0)	**<0.001**
CCL3 (pg/ml)	2.7 (0.0–7.0)	2.7 (0.0–6.7)	0.96
CCL5 (pg/ml *10^2^)	34.4 (15.0–79.6)	55.6 (31.2–115.6)	**0.005**
CCL18 (pg/ml *10^2^) hsCRP ≥50 % stenosis	769.0 (455.7–1266.0) 1.4 (0.7–3.1) 162 (24.5 %)	941.4 (529.9–1272.1) 1.8 (1.1–4.1) 41 (80.4 %)	0.23 0.07 **<0.001**

Values are presented as numbers (%), means ± standard deviation or medians (IQR). Significant *p*-values are in bold. *BMI* body mass index, *CCL* CC chemokine ligand, *hsCRP* high-sensitive C‑reactive protein

**Table 5:** Multivariable logistic regression analysis to predict for event

	OR	95 % CI	*p*-value
*Model 1*			
Lg10 CCL3	0.81	0.41–1.60	0.54
Lg10 CCL5	1.71	1.07–2.73	**0.03**
Lg10 CCL18	1.33	0.45–3.98	0.61
*Model 2*			
Lg10 CCL3	0.88	0.43–1.78	0.71
Lg10 CCL5	1.60	1.01–2.52	**0.046**
Lg10 CCL18	1.35	0.45–4.10	0.59
*Model 3 (CT ≥50 %)*			
Lg10 CCL3	0.96	0.44–2.07	0.91
Lg10 CCL5	1.35	0.88–2.06	0.17
Lg10 CCL18	1.46	0.45–4.72	0.53
*Model 4 (involvement score)*			
Lg10 CCL3	0.75	0.35–1.59	0.45
Lg10 CCL5	1.62	1.03–2.57	**0.04**
Lg10 CCL18	1.39	0.45–4.34	0.57

Model 1: corrected for age and gender; Model 2: corrected for age, gender, diabetes, BMI, smoking, family history, systolic blood pressure and total cholesterol; Model 3: corrected for age, gender, diabetes, BMI, smoking, family history, systolic blood pressure, total cholesterol and ≥50 % stenosis on CCTA; Model 4: corrected for age, gender, diabetes, BMI, smoking, family history, systolic blood pressure, total cholesterol and CCTA involvement score. *CAD* coronary artery disease, *CCL* CC chemokine ligand, *OR* odds ratio, *CI* confidence interval

## Discussion

In the present study, we investigated the association of CCL3, 5 and 18 with the severity and extent of CAD, as well as their predictive value for the occurrence of primary cardiac events. The main finding was that CCL5 demonstrated independent value to predict the presence of significant CAD. CCL18 was significantly associated with calcium and involvement scores. In addition, CCL5 but not CCL3 and CCL18 provided prognostic value for the occurrence of an event during follow-up, independent of traditional clinical risk factors as well as extent of CAD.

Previously, CCL3, 5 and 18 serum levels have been linked to cardiovascular disease (secondary events in early follow-up, refractory UAP, and ACS) and have been shown to be expressed by atherosclerotic plaque. Also, CCL5 has been related to carotid plaque characteristics [18]. However, evidence on the association of CCL3, 5 and 18 with CAD as well as on the prognostic value for primary ACS is conflicting and limited. Previous studies on CCL3, 5 and 18 have focused on the post-ACS stage, pointing to different roles of CCLs with regard to cardiovascular disease. For instance, CCL5 and CCL18 have shown to be transiently elevated during ischaemia. Especially CCL5 has been strongly associated with platelet activation, and serum levels are altered only transiently in the acute phase of UAP [6]. In our study, CCL5 was independently associated with the presence of ≥50 % coronary stenosis, a well-known risk factor for the occurrence of a cardiac event [19, 20]. This is in contrast to a recent, smaller, study investigating the association of CCL5 to ≥50 % coronary stenosis based on conventional angiography [21]. In addition, CCL5 serum levels also correlated with the occurrence of cardiac events. This association was independent of traditional risk factors. This independent association remained even when including the extent of CAD, a known risk factor. However, when ≥50 % coronary stenosis was added to the model, the significant association disappeared. Possibly, CCL5 is elevated in case of coronary obstruction, and not with extensive CAD only. Conceivably, CCL5 serum levels reflect platelet activation status [6] which could become manifest in obstructive CAD only. Therefore, CCL5 might be promising as an additional risk factor candidate before cardiac imaging diagnostics are applied. However, the value of CCL5 as ACS prognosticator in this study seems limited. Hypothetically, CCL5 might have potential as a therapeutic target as pharmacological inhibition of CCL5 could help to reduce plaque formation. Moreover, previous research showed that CC chemokine receptor 5 deficiency protected men from early myocardial infarction [22], and mice from early plaque formation [23]. On the other hand, previous studies showed a lack of association of CCL5 with future cardiovascular events [10, 11], another even showing a negative association [24]. Regarding these contrasting findings, at present the clinical implications of our findings might be small. We cannot conclude on causality, since we only have described associations. It is possible that CCLs might be elevated in case of an inflammatory state without a causative relationship with coronary atherosclerosis. For example, also hsCRP was associated with CAD.

CCL18 showed a particular association with both the coronary calcium score and the segment involvement score. This might be explained by the fact that CCL18 can directly stimulate fibrosis [4, 25], which might have an effect on calcification and more extensive plaque formation. Although CCL18 was not associated with events, it might be associated to the underlying mechanisms such as atherosclerosis progression. CCL18 showed a clear univariable association with extent of CAD as measured by coronary calcium score and involvement score. However, in multivariable analysis, CCL18 was neither independently associated with CAD (not for calcium, segment involvement score or obstructive CAD), nor with the occurrence of events. This is in contrast to the results of other prospective studies, in which CCL18 appeared to be an independent predictor of secondary cardiovascular events in the immediate aftermath of AMI or UAP [8, 26]. In a stable chest pain population, however, CCL18 seems to be dependent on calcification and plaque mass, but not predictive for future events.

The fact that we did not find an association between CCL3 and CAD and/or events, might seem contradictive to previous publications. For instance, higher CCL3 (and CCL5) concentrations were identified in patients with acute myocardial infarction as compared with controls [7]. Also, CCL3 (as well as CCL5 and 18) was independently associated with short-term mortality during follow-up in ACS patients [8]. Importantly, we measured CCL3, 5 and 18 concentrations prior to events. Therefore, collectively the data on CCL3 seem to plead for a role for CCL3 in the acute phase of an ischaemic event, not in the aetiology of ACS. CCL3 seems predominantly ischaemia derived [7] and may be involved in post-ischaemia inflammation, possibly through neutrophil-induced recruitment of monocytes [27].

## Limitations

We studied a relatively large population of patients with chest discomfort, who underwent CCTA to rule out obstructive CAD. Although this population meets our objectives, with the current cohort size, the low event rate of this low-risk population might have limited the value of longitudinal analyses. The need for revascularisation may be biased by the baseline CCTA. Finally, blood was drawn only at baseline and not during follow-up, which precludes assessment of the dynamics of chemokine patterns prior to an event.

## Conclusions

In patients with stable chest pain, CCL5 and 18 were associated with coronary obstruction and extent of CAD including calcification, respectively. These data suggest CCL5 and 18 are relevant for CAD development. Furthermore, CCL5 appeared to be associated with the occurrence of future cardiac events. This implicates that CCL5 could be a useful marker to predict for CAD and its consequences.
